# Operative Outcomes for Cervical Degenerative Disease: A Review of the Literature

**DOI:** 10.5402/2012/165050

**Published:** 2012-01-16

**Authors:** Kazuya Nishizawa, Kanji Mori, Yasuo Saruhashi, Yoshitaka Matsusue

**Affiliations:** Department of Orthopaedic Surgery, Shiga University of Medical Science, Seta Tsukinowa-cho, Otsu, Shiga 520-2192, Japan

## Abstract

To date, several studies were conducted to find which procedure is superior to the others for the treatment of cervical myelopathy. The goal of surgical treatment should be to decompress the nerves, restore the alignment of the vertebrae, and stabilize the spine. Consequently, the treatment of cervical degenerative disease can be divided into decompression of the nerves alone, fixation of the cervical spine alone, or a combination of both. Posterior approaches have historically been considered safe and direct methods for cervical multisegment stenosis and lordotic cervical alignment. On the other hand, anterior approaches are indicated to the patients with cervical compression with anterior factors, relatively short-segment stenosis, and kyphotic cervical alignment. Recently, posterior approach is widely applied to several cervical degenerative diseases due to the development of various instruments. Even if it were posterior approach or anterior approach, each would have its complication. There is no Class I or II evidence to suggest that laminoplasty is superior to other techniques for decompression. However, Class III evidence has shown equivalency in functional improvement between laminoplasty, anterior cervical fusion, and laminectomy with arthrodesis. Nowadays, each surgeon tends to choose each method by evaluating patients' clinical conditions.

## 1. Introduction

Cervical degenerative disease can result in manifestations distinct from degenerative disease of extremities. The cervical vertebrae contain the spinal cord; its compression by means of deteriorated cervical spine may lead to a generalized debility that sometimes culminates in tetraparesis as well as significant pain. When symptoms do not respond to conservative treatment, surgical treatment is considered. The goal of surgical treatment should be to decompress the nerves, restore the alignment of the vertebrae, and stabilize the spine. Consequently, the treatment of cervical degenerative disease can be divided into decompression of the nerves alone, fixation of the cervical spine alone or a combination of both. In addition, it can be divided into anterior or posterior procedures in terms of approach to the cervical spine. 

The purpose of this paper is to review the features of the operative treatment of cervical degenerative disease and outline the advantages and disadvantages of each approach and technique.

## 2. Posterior Approach

Posterior approaches have historically been considered safe and direct methods for cervical compression myelopathy with favorable clinical outcomes without fatal complications. In general, posterior approaches are indicated to the patients with cervical compression with posterior factors, multisegment stenosis and lordotic cervical alignment.

### 2.1. Cervical Laminectomy

Laminectomy has been a commonly undertaken standard posterior procedure. Large case series from the 1960s and 1970s and earlier have supported the use of this technique. At present, laminectomy still remains as a viable consideration for the surgical management of cervical myelopathy.

The studies reviewed experiences with laminectomy for cervical spondylotic myelopathy described success rates ranging from 42 to 92% [1–5]. On the other hand, culminating results have raised criticisms about this procedure. Cervical laminectomy is effective to decompress cervical cord; however it sacrifices posterior components of cervical spine. In turn, a number of studies reported the development of postoperative kyphosis and instability of cervical spine. These studies suggested that the incidence of postlaminectomy kyphosis ranged from 14 to 47% [6–11].

Another postoperative concern after cervical laminectomy is postoperative laminectomy membrane. The formation of a postlaminectomy membrane has been postulated by authors in most laminectomy reviews and clinical series [12]. In a series of patients undergoing reoperation after cervical laminectomy, however, Herkowitz [13] reported that the postlaminectomy membrane did not compress the spinal cord and nerve roots. Despite the wide spread references to a postlaminectomy membrane, there is no evidence that such a lesion has any clinical significance in humans who undergo laminectomy, nor is there evidence of clinical deterioration secondary to a postlaminectomy membrane in the literature [12].

Many authors have attempted to compare various procedures for the surgical management of cervical myelopathy. Matsunaga et al. [10] reported postoperative kyphosis in 34% patients in the laminectomy group compared to 7% in the laminoplasty group; functional outcome was not addressed.

 Although postlaminectomy kyphosis may be frequently observed radiographically, it is less clear how it relates to the development of clinical symptoms. Thus far no study has clearly demonstrated a relationship between postlaminectomy kyphosis and deterioration in the quality of life of the patients [14]. Overall it appears that laminectomy in selected patients compares favorably to alternative strategies.

### 2.2. Cervical Laminoplasty

Cervical laminoplasty was described in the 1970s as an alternative to laminectomy in patients with cervical myelopathy [15]. Laminoplasty permits expansion of the cervical canal with multisegments while preserving a dorsal laminar cover, which prevents the development of postlaminectomy membrane formation and/or postoperative kyphotic deformity. Several techniques of laminoplasty, such as the “open-door,” the midline “French-window,” and the “Z-plasty” techniques have been established; however there is no statistical difference of postoperative outcome among these techniques [12].

Many studies have demonstrated the effectiveness of laminoplasty. Although there are a variety of scales for the assessment of neurological function, the authors of most series used the Japan Orthopaedic Association (JOA) scoring system since laminoplasty was developed in Japan as an alternative to laminectomy [12]. Using the JOA scale score, the average recovery rate was 55 to 65%. Several studies corroborated clinical improvement maintained over 5 and 10 years [16–20]. The functional improvement after laminoplasty may be limited by duration of symptoms, severity of stenosis, severity of myelopathy, and poorly controlled diabetes as risk factors [21]. There is conflicting evidence regarding patient age with one study [22] citing age as a risk factor, but the others [23, 24] not demonstrating this result.

Laminoplasty has been associated with reduced range of motion (ROM) of the cervical spine [16, 17, 20, 25–27]; however it has not always signified poor outcome. Indeed, Kihara et al. [26] reported that mean JOA scale score of the patients with cervical myelopathy significantly improved by laminoplasty, while ROM of the cervical spine decreased from 36.9 to 29.1. Saruhashi and colleagues [17] reviewed 30 patients who underwent “French-window” laminoplasty for cervical spondylotic myelopathy. Patients were followed up for 5 years, and JOA scale score improved significantly from a preoperative average of 8.8 to a postoperative average of 11.9. Simultaneously, alignment deteriorated in some (loss of 12.5 degrees) and stabilized in others (gain of 1.1 degrees). In comparing these 2 groups, the authors observed no significant difference in mean JOA scale score. Shiraishi et al. [28] compared skip laminectomy to open-door laminoplasty for the treatment of cervical myelopathy. There is no significant difference in these groups in terms of JOA scale score recovery with minimum 2-year followup, while ROM was 98% preserved in the skip laminectomy group compared to 44% in the laminoplasty group.

As one of the complications of posterior approach (laminectomy and laminoplasty), nuchal and shoulder pain (so-called axial pain) have been reported. Postoperative axial pain was observed 5.2 to 61.5% [29]. To prevent this complication, several modifications were tried. Preservation of muscular attachment to C2, restoration of ligamentum nuchae, and preservation of its attachment to C6 or C7 have been reported [30]. Accordingly, these modifications might decrease postoperative sever axial pain.

As another relatively frequent complication after cervical decompression surgery, postoperative transient segmental motor palsy on an upper limb has been reported [16, 18, 20, 31, 32]. Among postoperative segmental motor palsies which originate from C5–C8 monosegmental or multisegmental lesion, C5 segmental palsy is the most common known as “C5 palsy.” The incidence of C5 palsy has been reported previously with the average of 4.6%, varied from 0% to 30.0% [33]. To avoid this complication, pathomechanisms of this paralysis and/or a selection of surgical procedure have been discussed elsewhere [34–37]. Several factors such as local reperfusion injury in the spinal cord [34, 35], the excessive posterior shift of the spinal cord [38], and tethering of the nerve root [28, 39] have been implicated in this palsy; however controversies still remain, and gold standard procedure for the prevention of C5 palsy has not been established yet.

Consistent with cervical laminectomy, the development of postoperative kyphosis after cervical laminoplasty has been reported [12]. However, it was less frequently than cervical laminectomy. Indeed, the incidence of postoperative kyphosis after laminoplasty was reported 2–28% [12, 18, 40], while that of laminectomy was 14–47% [6–11].

### 2.3. Cervical Laminectomy/Laminoplasty with Fusion

Laminectomy/laminoplasty has been the traditional and safety procedure to decompress spinal cord in patients with cervical myelopathy. On the other hand, because of concern over deterioration from the long-term effects of resultant segmental instability and/or kyphosis, alternative to cervical laminectomy/laminoplasty has been developed. Laminectomy/laminoplasty with posterior fusion allows posterior canal expansion and spinal stability. This modification theoretically avoids problems associated with laminectomy/laminoplasty alone. Furthermore, with the development of internal fixation devices, it may allow reduction of kyphosis to lordosis, thereby broadening indications for posterior spine surgery in the treatment of myelopathy. Several studies support the use of laminectomy/laminoplasty with fusion for the treatment of cervical myelopathy [41–45]. It has been reported that 70–95% of patients show postoperative neurological improvement.

 The technique of fusion has evolved. Initially it was performed with on-lay posterolateral bone grafting into laminoplasty troughs and/or into facets. Documentation of fusion success was inadequate in all studies, but there appeared to be high rates of failures. The use of lateral mass wires, screws-rod or screw-plate constructs theoretically resulted in more stable constructs and higher fusion success. On the other hand, complications related to misplaced screws, hard ware failure with loss of alignment, radiculopathy, screw malposition, and the need for a repeated operation have been reported [46].

### 2.4. Cervical Laminoforaminotomy

The first documented description of the surgical treatment of a herniated cervical disc was by Spurling and Scoville [47], who provided a description of a posterior approach to the cervical spine for treatment of a herniated cervical disc via a laminoforaminotomy procedure. This description of laminoforaminotomy predated the initial reports of anterior cervical discectomy by Clowrd [48, 49] and Smith and Robinson [50] by 10 years. Posterior laminoforaminotomy is recommended as a surgical treatment option for symptomatic cervical radiculopathy resulting from either a soft lateral disc displacement or spondylosis with resultant foraminal stenosis caused either by a herniated disc, osteophyte, or both. Advantages to posterior laminoforaminotomy include sparing the motion segment. Furthermore, there is the theoretical advantage that adjacent segment disc degeneration, which is becoming increasingly recognized after anterior cervical fusion, is unlikely to occur in patients undergoing laminoforaminotomy. Several studies support the use of laminoforaminotomy for treatment of cervical radiculopathy [51]. They show consistently that 75–98% of patients show postoperative neurological improvement. Further surgery for recurrent root symptoms was performed on approximately 6% [52, 53].

## 3. Anterior Approach

In general, anterior approaches are indicated to the patients with cervical compression with anterior factors, relatively short-segment stenosis without spinal canal stenosis in other regions, and kyphotic cervical alignment.

### 3.1. Anterior Cervical Interbody Fusion

The most frequently cited technique for anterior discectomy and fusion is the one described by Emery et al. [54]. Decompression involves removal of the soft disc and/or osteophyte from the compressed neural elements so they no longer impinge on the nerves. Restoration of alignment of cervical spine includes restoration of the disc space height and neural foraminal height as well as the normal angle between the vertebrae. Stability involves elimination of motion of the motion segment. Therefore, a fusion technique can be used, provided it incorporates a structural support to replace the disc and that a stable fusion of the vertebrae is acquired.

The population of the anterior approach for discectomy and fusion has increased because this approach avoids exposure of the spinal canal and results in less soft tissue damage [55].

The common surgical technique to treat cervical spondylotic myelopathy is removal of the damaged disc(s) and/or osteophyte with bone transplantation. The fusion rate for single-level fusions ranged from 89 to 99% [56, 57] and for dual-level fusions ranged from 72 to 90% [56–59]. These studies described success rates ranging from 75 to 96% for single- or dual-level fusions. For the multilevel fusions, the fusion rate was decreased compared with the single- or dual-level fusions [58]. The success rate for the multilevel fusions ranged from 60 to 88% [60].

Most frequently reported problems include postoperative pain, wound hematoma, infection, pelvic fracture, nerve palsy, and chronic donor site pain with the incidence of an average of 2.4% [61]. In a study that specifically looked at donor site pain, no less than 90% of patients complained of donor site pain [62]. By contrast it is not necessary to pay attention to donor site pain through the use of interbody cages. They provide initial stability, and by filling the disc space, require less structural bone graft. However, when we look at fusion rates, iliac crest autograft is superior to interbody cages [63].

Adjacent disc degeneration after anterior cervical interbody fusion is also a relatively common complication. The incidence of adjacent disc degeneration after cervical anterior cervical interbody fusion has been reported as 11–33% [64, 65]. Patient-caused symptomatic adjacent disc degeneration sometimes requires additional surgery on the cervical spine. In long-term follow-up studies, the rate of revision surgery has been reported to be 6.3–16.9% [64, 66]. Hilibrand et al. [66] reported that the C5-6 and C6-7 discs had a high risk of symptomatic anterior disc degeneration after cervical anterior cervical interbody fusion. Komura et al. [64] described that anterior disc degeneration occurred less frequently among patients in whom C5-6 and C6-7 were fused than among those in whom C5-6 or C6-7 was left at an adjacent level.

## 4. Cervical Disc Arthroplasty

The first paper related to cervical disc arthroplasty was published in 2002 [67]. The theoretical advantage of arthroplasty should be that reconstruction of a failed intervertebral disc with functional disc prosthesis. This technique should preserve motion segment; thereby protect the adjacent level discs from the abnormal stresses associated with fusion. Since 2002, the results of several RCTs have been published. In all of these RCTs, the proponents of cervical disc arthroplasty stated that its rationale was to decrease the likelihood of adjacent-segment degeneration [68]. However, no study has specifically compared outcome with respect to anterior disc degeneration after cervical disc arthroplasty or fusion and there is no clinical evidence of reduction in anterior disc degeneration with the use of cervical disc arthroplasty [68].

## 5. Summary

To date, several studies were conducted to find which procedure is superior to the others for the treatment of cervical myelopathy. Laminoplasty was compared with other techniques in several studies. Wada et al. [69] compared subtotal corpectomy to open-door laminoplasty. The JOA scores improved in both groups; however, the incidence of moderate or severe pain was greater with laminoplasty, and ROM was better preserved with corpectomy. Yonenobu et al. [32] reported on 83 patients who underwent French-window laminoplasty and 41 who underwent anterior cervical fusion. The JOA scores improved in both groups; however, the complications were higher with anterior cervical fusion due to graft complications. Review of the current, peer-reviewed literature did not resolve whether an anterior or a posterior surgery would have better short- and long-term results [51].

There is no Class I or II evidence to suggest that laminoplasty is superior to other techniques for decompression. However, Class III evidence has shown equivalency in functional improvement between laminoplasty, anterior cervical fusion, and laminectomy with arthrodesis [21].

 Nowadays, each surgeon tends to choose each method by evaluating patients' clinical conditions.
